# Inferring the evolution of reproductive isolation in a lineage of fossil threespine stickleback, *Gasterosteus doryssus*

**DOI:** 10.1098/rspb.2024.0337

**Published:** 2024-04-17

**Authors:** Raheyma Siddiqui, Samantha Swank, Allison Ozark, Franklin Joaquin, Matthew P. Travis, Caleb D. McMahan, Michael A. Bell, Yoel E. Stuart

**Affiliations:** ^1^ Department of Biology, Loyola University Chicago, Chicago, IL, USA; ^2^ Committee on Development, Regeneration, and Stem Cell Biology, University of Chicago, Chicago, IL, USA; ^3^ Department of Biological and Biomedical Sciences, Rowan University, Glassboro, NJ, USA; ^4^ Field Museum of Natural History, Chicago, IL, USA; ^5^ University of California Museum of Paleontology, Berkeley, CA, USA

**Keywords:** anagenetic speciation, *Gasterosteus aculeatus*, punctuated equilibrium, time series, ephemeral speciation, ecological speciation

## Abstract

Darwin attributed the absence of species transitions in the fossil record to his hypothesis that speciation occurs within isolated habitat patches too geographically restricted to be captured by fossil sequences. Mayr's peripatric speciation model added that such speciation would be rapid, further explaining missing evidence of diversification. Indeed, Eldredge and Gould's original punctuated equilibrium model combined Darwin's conjecture, Mayr's model and 124 years of unsuccessfully sampling the fossil record for transitions. Observing such divergence, however, could illustrate the tempo and mode of evolution during early speciation. Here, we investigate peripatric divergence in a Miocene stickleback fish, *Gasterosteus doryssus.* This lineage appeared and, over approximately 8000 generations, evolved significant reduction of 12 of 16 traits related to armour, swimming and diet, relative to its ancestral population. This was greater morphological divergence than we observed between reproductively isolated, benthic-limnetic ecotypes of extant *Gasterosteus aculeatus*. Therefore, we infer that reproductive isolation was evolving*.* However, local extinction of *G. doryssus* lineages shows how young, isolated, speciating populations often disappear, supporting Darwin's explanation for missing evidence and revealing a mechanism behind morphological stasis. Extinction may also account for limited sustained divergence within the stickleback species complex and help reconcile speciation rate variation observed across time scales.

## 1. Background

Darwin's sharpest critique of his own theory on the origin of species was that morphological transitions between ancestral and descendent species had not been observed in the fossil record [1]. Darwin had proposed that the Earth's biological diversity arose though the gradual accrual of small differences between lineages over many generations under natural selection [2]. 'Why then', Darwin asked, ‘is not every geological formation … full of … intermediate links? … this, perhaps, is the most obvious and gravest objection which can be urged against my theory’ ([2], p. 280). Vexed, Darwin devoted chapter IX of *On the Origin of Species* (‘On the Imperfection of the Geological Record’) to explain the missing transitions between fossil species [2]. He cited the practical problem of missing fossil data, but also proposed a biological explanation: ‘there is reason to suspect … that … varieties are generally at first local; and … [they] do not spread widely … until they have been modified … to some considerable degree … the chance of discovering … the early stages of transition between any two forms, is small … ’ ([2], p. 298). Thus, Darwin reconciled natural selection among small variants with the absence of fossilized gradual transitions: detection is unlikely because divergence was localized to small populations. Morphological change is detected, therefore, only as a sudden appearance after the new species spreads. As such, Darwin anticipated peripatric speciation [3] and punctuated equilibria [4]. Testing this speciation model is difficult because one needs a fossil deposit that contains a chain of ancestral and descendent populations, a finely resolved stratigraphic record to capture graded change over generations [5], and some way to infer whether observed phyletic divergence is associated with the evolution of reproductive isolation.

We present a rare confluence of these requirements by comparing gradual multivariate evolution in a well-resolved lineage of fossil stickleback fish (*Gasterosteus doryssus*) to multivariate divergence between reproductively isolated benthic-limnetic ‘species pairs’ of the closely related, extant threespine stickleback (*Gasterosteus aculeatus*). We infer gradual evolution of reproductive isolation in a peripheral fossil population and discuss the implications of this fossil case for ecological speciation and punctuated equilibrium.

### (a) The modern comparison: species-pair lakes

The so-called ‘species-pair’ lakes each support a benthic and a limnetic ecotype of *G. aculeatus*. The pairs have evolved independently in at least five lakes in British Columbia, Canada [6–9]. The ecotypes are consistently divergent in shape, size, feeding morphology and armour [6,10–12] and have evolved substantial reproductive isolation within each lake [7,9,13–16]. Within lakes, the ecotypes do occasionally hybridize [12,16–18], but hybrids have poor feeding and growth performance [19,20], and low fitness [21]. This results in genetic divergence maintained at ecologically relevant loci.

### (b) Fossil stickleback evolution

**Figure 1.**
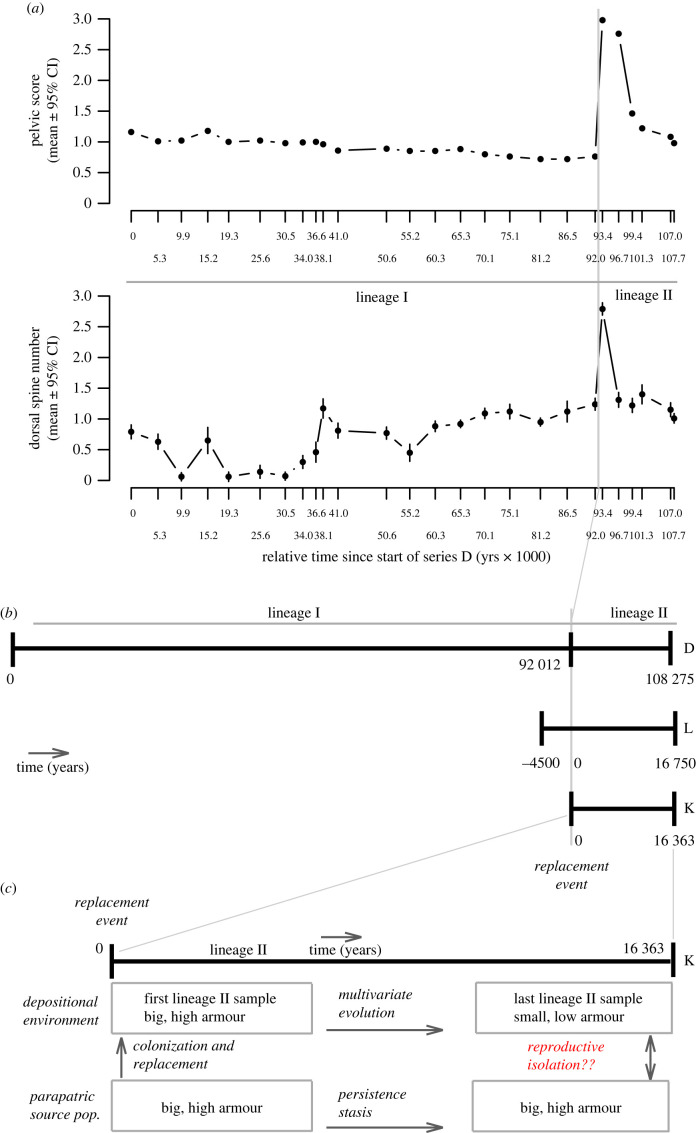
(*a*) Variation of pelvic score and dorsal spine number means through time (with 95% confidence intervals if visible), showing the transition in phenotype at the replacement event. Data from appendix 1 of Bell *et al*. [24]. (*b*) Stratigraphic correlation in years for fossil series D, L and K [24–26], all sampled from the same Quarry D palaeolake bed. (*c*) Proposed speciation scenario: lineage II colonizes the depositional environment, replacing lineage I in less than 125 years. During the next approximately 16 000 years, lineage I evolved reduction in size, armour, swimming and trophic traits. By the end, is lineage II reproductively isolated from its armoured source population?

*Gasterosteus doryssus* has been sampled through approximately 108 000 years of diatomite sediment in Quarry D [22], a single 10-million-year-old Miocene stratigraphic section in the now-dried Lake Truckee, Nevada, USA [23]. Three fossil time series called D, L and K were collected from this one Quarry D section (figure 1) [24–26]. During the first approximately 92 000 years, the *G. doryssus* population had low armour, with no lateral plates, and usually small pelvic structures and zero to one dorsal spine (hereafter, lineage I; figure 1). However, a single, highly armoured specimen with a full pelvic skeleton and three dorsal spines was found in each of five of the seven samples from the first approximately 30 500 years of the lineage I series D fossil sequence [24]. These rare high-armoured forms probably represented arrivals from at least one armoured population living elsewhere within the broader lake basin, but outside of the Quarry D depositional environment [22,23]. Moreover, the fourth sample in this time series had seven specimens that had both a full pelvic skeleton and three dorsal spines, along with 63 specimens with the typical lineage I, low-armour phenotypes [24]. The association between dorsal spine and pelvic phenotypes in this sample is not random (analysis below); having three dorsal spines was perfectly associated with having a full pelvis. In extant *G. aculeatus,* dorsal spine number and pelvic structure often do not map to the same genes [27–30]; but see [31]. Thus, if a similar genetic basis underlies trait divergence in both ancient *G. doryssus* and extant *G. aculeatus* (see Methods as well as [26,31–37]), then any interbreeding among high- and low-armoured *G. doryssus* in the depositional environment should have produced a random association between the two armour traits. The observed, non-random association in this fourth sample of series D suggests that reproductive isolation existed between high- and low-armoured sticklebacks in the basin [23].

At approximately 92 000 years, an abundant, high-armoured stickleback with a full pelvic skeleton and three dorsal spines (hereafter lineage II) replaced the previously abundant, low-armoured lineage I within a few decades [25,26] (figure 1). The single L-series sample that contained the transition from the low to high-armoured stickleback lineage included 40 specimens with fewer than three dorsal spines and reduced pelvic structures in the earlier part of the sample and 26 specimens with three dorsal spines and a full pelvis later in the sample [25]. No specimens in this sample had a high spine number and a reduced pelvis or vice versa. This again suggests reproductive isolation between low-armoured lineage I individuals and fish from high-armoured populations nearby. However, if hybrids were rare, the absence of intermediates may be a consequence of sample size: of 441 L-series specimens sampled from the 3000 years before the high-armoured form appeared *en masse*, 14 specimens (3.2%) had full pelvises but reduced dorsal spines, and two specimens (less than 1%) had three dorsal spines but a reduced pelvis. These mixed-armour fish suggest that lineage I did have contact with armoured populations but only low levels of hybridization, similar to the modern, reproductively isolated species pairs [16,18,21].

Local extinction of low-armoured lineage I is reflected by the L-series sample approximately 250 years after the appearance of high-armoured forms. This sample had 87 specimens, all with complete pelvic skeletons and all but five with three dorsal spines. This almost instantaneous ecological ‘replacement’ may have been caused by a change in the prey community [38] and an increase in lake productivity and depth, which connected the depositional environment to other parts of the basin [39,40] thereby changing selection to favour armour [41]. After the replacement event, however, water levels in the depositional environment dropped again [40]. This apparently restored the original low-armour selection regime because lineage II began evolving via natural selection a low-armour phenotype resembling that of lineage I [25,41]. By approximately 16 500 years after the replacement event, lineage II in this depositional environment had evolved significant reduction in traits related to armour, swimming and feeding [24–26] (electronic supplementary material, figure S1).

### (c) Phyletic evolution and speciation

Does this gradual morphological evolution of low armour by lineage II reveal the early stages of speciation from its parapatric, high-armoured ancestral source (figure 1)? The infrequency of armour intermediates in lineage I samples despite contact with high armoured populations (described above) suggests that the evolution of low-armour in lineage II should have also generated reproductive isolation. Yet the neontologist's niggling question remains: is the observed change by lineage II in the depositional environment indeed indicative of reproductive isolation from its parent population and the gradual formation of a new biological species ([42], pp. 62–68)? After all, hybridization may have been rare but still high enough to inhibit reproductive isolation.

To investigate this question, we compared morphological evolution by lineage II to morphological differences between reproductively isolated benthic and limnetic ecotypes in five species-pair lakes. Recall that fieldwork and experiments have shown that multivariate morphological divergence between benthics and limnetics is correlated with ecological partitioning, fitness differences, mate choice and reproductive isolation. For example, these benthic and limnetic stickleback contrast for diet—larger benthic invertebrates versus zooplankton (e.g. [6])—in the same way that fossil specimens from the earliest and later lineage II samples appear to have differed [38]. In addition, the benthic-limnetic species pairs are the only type that coexist within single lakes. They maintain their ecological and phenotypic divergence and reproductive isolation as long as the environment is relatively undisturbed [43,44] and therefore meet the criteria of several species concepts [45].

We propose that if phenotypic divergence observed between the first and last samples of *G. doryssus* lineage II approaches or exceeds phenotypic divergence between benthic-limnetic pairs, then we can infer that gradual evolution by lineage II was enough to generate reproductive isolation from its source population (figure 1), representing a rare observation of early speciation in the fossil record.

## 2. Methods

### (a) Fossil specimen data

To quantify morphological divergence in the fossils, we compiled published data from the D [24], the L [25] and the K series [26,46] (figure 1 for stratigraphic correlations among series). Each series comes from quarry D (i.e. the same palaeolake bed [22]). Series D consisted of 26 samples spaced at approximately 5000-year intervals over an estimated 108 275 years (figure 1). Six traits were measured by Bell *et al*. [24] from D samples: standard length, pelvic score, and the number of pre-dorsal pterygiophores, dorsal spines, anal-fin rays and dorsal-fin rays. A single exposure in the upper portion of series D contains series L and K (figure 1). Series L is a nearly continuous stratigraphic section spanning approximately 4500 years before to approximately 16 500 years after the replacement of lineage I by lineage II. Three armour traits were measured by [25] for series L: pelvic score, number of dorsal spines and number of touching pre-dorsal pterygiophores.

Our analysis focuses on series K [26,46] because 16 traits were measured for K (see electronic supplementary material, table S1 and table S2 for sample sizes, which ranged from 12 to 67 across samples and traits). This allows estimates of multivariate divergence in traits reflecting not just defense, but also swimming, feeding and body size. These traits are also divergent between benthic and limnetic ecotypes in the species-pair lakes [6,7,13]. Whether, when and how any single trait adds to reproductive isolation in threespine stickleback is uncertain (see [16]). Body size, however, is a known correlate of mate choice and assortative mating in the stickleback species pairs [7,13,47,48]. Moreover, the ecological relevance of the multivariate trait set makes it more likely that we will have measured traits related directly to assortative mating or indirectly via trait correlations or selection against hybrid phenotypes. Series K is from 18 narrow time intervals taken at roughly 1000-year periods from exactly the same section and exposure as series L. Series K spanned approximately 16 363 years, starting at the replacement horizon, when lineage I and lineage II specimens occurred within a single sample. We removed lineage I fish from this sample for morphological analysis.

Finally, we note that a highly armoured population existed in Quarry E (*sensu* [22,49]), 1.7 km from Quarry D [23]. Quarry E appears to have been deposited nearshore, per its abundant terrestrial plant fossils and thick clastic layers embedded with diatomite [23]. This site contrasts Quarry D's open water habitat [23,40]. Quarry E was dated to roughly the same time as the K series [49], so at least one nearby armoured population existed concurrently with lineage II appearing and diverging in Quarry D (figure 1*c*).

### (b) Extant specimen data

We measured the same 16 traits that had previously been scored for the K-series in benthic and limnetic ecotypes from five species-pair lakes (table 2; see electronic supplementary material, table S3 for sample sizes, which ranged from 22 to 69 per lake-ecotype combination across traits). Specimens were collected by D. Schluter's laboratory (University of British Columbia) from Enos Lake in 1988 and from Emily, Little Quarry, Paxton and Priest Lakes in 2018. In 2019, we stained these specimens for bone using Alizarin Red. Standard length as well as pelvic-spine lengths were measured with calipers. We used a dissection microscope to count dorsal spines, pelvic spines, dorsal-fin rays and anal-fin rays. Pelvic girdle and ectocoracoid lengths were measured from ventral photographs (Canon EOS Rebel T7, Tamron 16–300 mm MACRO lens). Lateral X-rays were used to measure lengths of the dorsal spines, pterygiophore anterior to the third spine, cleithrum and premaxilla ascending branch. We also counted the number of pterygiophores anterior to the pterygiophore holding the third spine and of abdominal and caudal vertebrae. Caudal vertebrae included the first vertebra with the haemal spine contacting an anal fin pterygiophore and all posterior [50]. X-rays were taken with an AXR Hot Shot X-ray machine (Associated X-ray Corporation) at the Field Museum of Natural History. Specimens were exposed at 35 kV and 4 mA for 7 to 10 s. We scanned individual fish images using the B&W Negatives setting on an Epson Perfection 4990 Photo flatbed at 2400 dpi. Measurements from photographs and X-rays were taken with FIJI [51] and its plugin ObjectJ (https://sils.fnwi.uva.nl/bcb/objectj/).

### (c) Analysis

Analyses were conducted in R [52]. Code and data to recreate analyses are at https://doi.org/10.5061/dryad.mpg4f4r53 [53].

#### (i) Tests for hybridization between high- and low-armoured *G. doryssus*

Many freshwater-adaptive genetic variants in stickleback are ancient [36] and present as standing genetic variation in modern marine populations [33,54,55]. They have been used in parallel during adaptation of countless populations that have colonized fresh water [31,32,34,35,37], including across species separated by tens of millions of years (e.g. *G. aculeatus* and the ninespine stickleback *Pungitius pungitius*; timetree.org). Pelvic girdle and dorsal spine phenotypes are at least partially controlled by unlinked genes in *G. aculeatus* [28,–31,56,57], and we assume similar genetic architecture for the closely related *G. doryssus.* Therefore, we hypothesized that pelvic girdle and dorsal spine phenotypes were free to vary independently in a panmictic population of *G. doryssus* that is polymorphic for these traits. This assumption of shared architecture is supported by morphological evidence. The major gene for pelvic reduction in *G. aculeatus* and *P. pungitius* is *Pitx1* [28], [58]. This mode of pelvic reduction results in asymmetrical pelvic vestiges, which are also observed in lineage II *G. doryssus*, suggesting shared use of *Pitx1* [26]. Moreover, lineage II immediately began evolving loss of dorsal spines (electronic supplementary material, figure S2) [26,41], while pelvic score did not begin reduction for approx. 1500 generations (electronic supplementary material, figure S2) [25,26], suggesting independent genetic bases.

Therefore, we used Fisher's exact test on samples where high- and low-armoured forms were collected together—Sample 4 of series D [24] and the replacement event sample in series L [25]—to test if a complete pelvis (pelvic score = 3) was significantly associated with having three dorsal spines, and conversely, whether pelvic reduction (pelvic score < 3) was significantly associated with having fewer than three dorsal spines. Significant association would suggest that hybridization is rare and indicate at least some reproductive isolation.

#### (ii) Data checks and preparation, outlier analysis and size correction

For remaining analyses, we used data from the K series and species pairs. We randomly selected one pelvic spine from each extant specimen because only one spine was measured in the fossils. To check for outliers, we calculated within-group means and standard deviations for each trait separately for fossil specimens (pooled across samples) and for extant specimens (pooled across lakes). We noted potential outliers as trait values greater than 3.5 s.d. from the mean (assuming a normal distribution). We deemed 3.5 s.d. to be a reasonable threshold for detecting errors without excluding biologically relevant values. We checked whether these potential outliers were a result of data entry error and corrected them if they were. We turned the remaining outlier trait values to NAs. We size-corrected only continuous traits, as they varied with size, unlike count traits that are fixed during early development. We regressed each trait on standard length using a mixed-model regression [59]. We size-corrected the fossil and extant samples separately because they had different histories and ecologies. Separate size-corrections allowed different allometric relationships rather than forcing a single correction. With category-specific, size-corrected values, we could then measure magnitudes of divergence within each category before comparison.

#### (iii) Trait-by-trait divergence

We compared 16 traits between the first and last series K samples. We calculated the *U*-statistic for each trait with a Wilcoxon rank-sum test (*wilcox.test*, *stats* R package). *p*-values < 0.003 were considered significant (multiple test correction; *α* = 0.05 / 16 traits = 0.003). We calculated a common-language effect size as *U* divided by the product of the sample sizes. Common language effect sizes near 0 or 1 reveal consistent differences in trait value between samples. We calculated the same statistics contrasting benthic and limnetic ecotypes from each species pair.

#### (iv) Multivariate divergence

We calculated the multivariate Euclidean distance, *d*, between the first and last fossil samples using all traits. We bootstrapped an error estimate about *d* by resampling each temporal fossil sample with replacement 99 times, recalculating *d* each time. We used permutation to test whether *d* was significantly larger than expected, shuffling sample identity (first versus last) 99 times and recalculating *d*. We calculated the same statistics between benthic and limnetic ecotypes from each species pair.

The scale for standard length is an order of magnitude larger than the other traits. Normally, a log transformation helps rescale, but we have count and continuous traits for which zeroes are meaningful due to evolutionary loss. Therefore, to understand the influence of standard length on estimates of *d*, we excluded standard length and re-ran the analyses (electronic supplementary material).

#### (v) Comparison of fossil *d* to benthic-limnetic *d*

To compare fossil and species-pair *d* values statistically, we used the *t.test* function (*stats* R package; *two-sided, paired = FALSE*) to test whether the bootstrap distribution of fossil *d* overlapped with species-pair distributions.

#### (vi) Timing of morphological divergence

We quantified how long it took for lineage II fossil divergence to attain levels of benthic-limnetic divergence by calculating *d* iteratively between the first K-series sample and each subsequent K sample. We calculated a boostrap distribution for each *d* and plotted when these distributions first overlapped with *d* for the least diverged species pair (a minimum time to ‘species-level’ reproductive isolation) and with mean species-pair *d*.

#### (vii) Correlated trait evolution

To visualize how traits change together during the accrual of reproductive isolation, we calculated a vector of temporal sample means for each trait. We then calculated pairwise Pearson correlation coefficients among vectors for all traits.

## 3. Results

### (a) Tests for hybridization between high- and low-armoured *G. doryssus*

In D-series sample 4, having three dorsal spines was perfectly associated with having a full pelvis (*n* = 70; seven specimens with full pelvises had three dorsal spines; 63 specimens with reduced pelvises had less than three dorsal spines; Fisher's exact test, *p* < 0.0001). Each of those 63 specimens had pelvic scores less than or equal to one; three of those 63 individuals had two dorsal spines, with the other 60 having zero to one dorsal spine. In the series L replacement event sample, having three dorsal spines was also perfectly associated with having a full pelvis (*n* = 66; 26 specimens with full pelvises had three dorsal spines; 40 specimens with reduced pelvises had less than three dorsal spines; Fisher's exact test, *p* < 0.0001). Thirty-seven of the 40 individuals with reduced pelvises had pelvic scores less than or equal to one; the other three had pelvic scores of 2.6, 2.4 and 1.6. Three different individuals had pelvic scores of one with two dorsal spines. A plot of dorsal spine and pelvic score reduction by Lineage II through time after the replacement event confirms that the mixed armour state (e.g. pelvic score = 3 and dorsal spine number < 3) is possible, and that the two traits can evolve independently (electronic supplementary material, figure S2).

### (b) Trait-by-trait divergence

**Figure 2.**
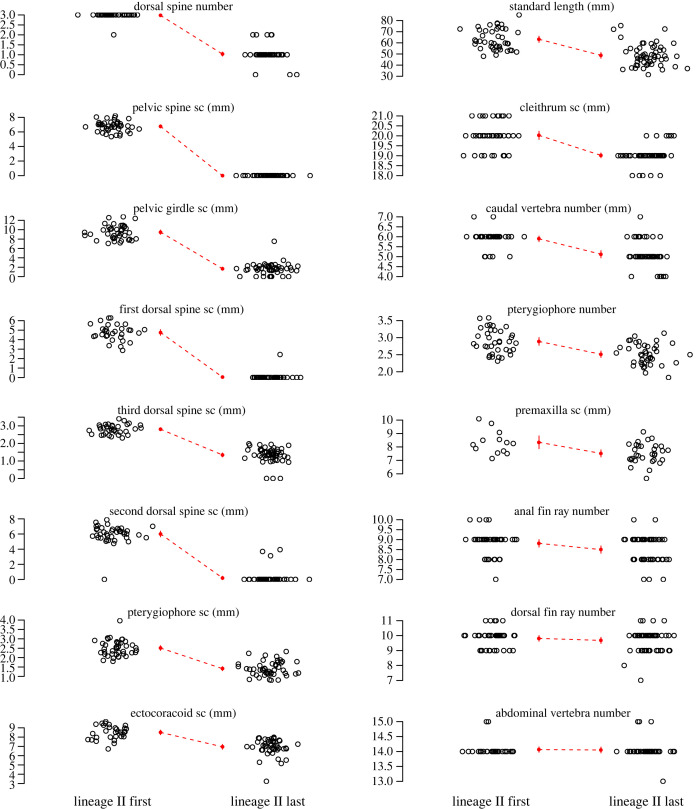
Divergence between first (earliest) and last (latest) lineage II samples. Open circles are data that have been jittered. Red points are sample means with 95% confidence intervals if visible. Traits are plotted in descending order by Common Language Effect Size, first column top to bottom, then second column top to bottom (table 1). ‘sc’ denotes size corrected trait lengths.

The first and last samples of lineage II were significantly different for 12 of 16 traits at the *p* < 0.003 level; a 13th trait was marginally non-significant with *p* = 0.005 (figure 2 and table 1). Within species-pair lakes, extant benthic and limnetic ecotypes differed significantly for four to 12 traits (mean across five pairs = 9.4, s.d. = 2.9) at the *p* < 0.003 level (electronic supplementary material, table S4).

**Table 1.** Earliest and latest samples of series K lineage II differed significantly for 12 of 16 traits (at Bonferroni-corrected *p* = 0.003 level). Median and mean differences are the earliest lineage II sample subtracted from the latest lineage II sample. *n* first and *n* last are the first and last sample sizes, respectively. The *U*-statistic is from a Wilcoxon rank-sum test with continuity correction. *p* is the probability that the null hypothesis of no difference is correct; bold values are statistically significant after multiple-test correction. Common language effect size is the proportion of pairwise comparisons between individuals from earliest and latest samples in which the individual from the last sample had a higher value than the individual from the first sample. Values close to zero indicate that individuals from the last sample have smaller trait values than individuals from the first sample. See electronic supplementary material, table S1 for trait codes. Differences of size-corrected (sc) traits are in mm.

trait code	median difference	mean difference	pooled variance	*n* first	*n* last	*U*-statistic	*p*-value	common language effect size
mds	−2.00	−1.96	0.32	42	55	2.50	**0**.**000**	0.00
lps.sc	−6.85	−6.81	0.50	42	49	0.00	**0**.**000**	0.00
tpg.sc	−7.68	−7.85	135	41	49	3.00	**0**.**000**	0.00
ds1.sc	−5.03	−4.88	0.61	31	50	0.00	**0**.**000**	0.00
ds3.sc	−1.46	−1.47	0.37	35	54	0.00	**0**.**000**	0.00
ds2.sc	−6.24	−5.93	1.04	40	53	28.0	**0**.**000**	0.01
lpt.sc	−1.12	−1.09	0.39	41	47	46.00	**0**.**000**	0.02
ect.sc	−1.49	−1.55	0.82	32	46	92.00	**0**.**000**	0.06
stl	−12.88	−14.45	9.49	43	55	318.50	**0**.**000**	0.13
mcv	−1.00	−1.01	0.56	42	55	321.00	**0**.**000**	0.14
mpt	−1.00	−0.77	0.56	39	48	346.00	**0**.**000**	0.18
pmx.sc	−0.34	−0.38	0.33	37	41	339.00	**0**.**000**	0.22
cle.sc	−0.80	−0.83	0.78	13	31	92.00	0.005	0.23
maf	0.00	−0.31	0.65	43	52	840.50	0.020	0.38
mdf	0.00	−0.14	0.70	43	53	1052.50	0.473	0.46
mav	0.00	−0.02	0.29	31	40	612.00	0.857	0.49

### (c) Multivariate divergence

**Figure 3.**
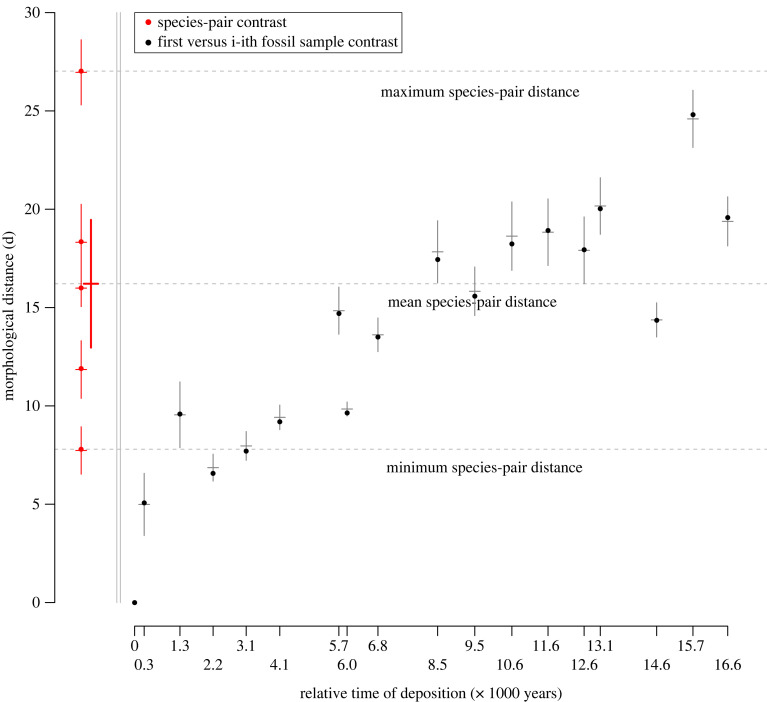
Multivariate divergence (*d*) among lineage II fossil samples and benthic-limnetic species pairs. Black circles are the multivariate distance *d* between the first lineage II sample and each subsequent sample. Red circles are *d* for each species pair. Horizontal and vertical lines about each point are the mean and standard deviation, respectively, of 99 bootstrap estimates of *d*. The bold red cross is the grand mean and standard error of *d* across the species pairs. The species pair lakes from top to bottom are Little Quarry, Enos, Paxton, Priest and Emily. The rightmost black circle represents *d* between the first and last fossil samples.

Multivariate *d* between the first and last lineage II fossil samples was larger than benthic-limnetic *d* for four of the five species pairs (all *p* < 0.0001; figure 3; table 2). Removing standard length, fossil *d* was greater than all five species-pair *d* values (electronic supplementary material, figure S3), confirming that fossil divergence is large. Permutation distributions never contained observed *d* values (electronic supplementary material, figure S4), indicating significant multivariate differences between fossil samples and between ecotypes. Bootstrap distributions about *d* were narrow relative to the differences between samples/ecotypes, suggesting robustness to sampling error (electronic supplementary material, figure S4).

**Table 2.** Welch two sample *t*-tests comparing the bootstrap distribution of *d* for the first and last fossil samples against bootstrap distributions for each species pair. Mean *d* is the mean of the bootstrapped species-pair distribution. Mean bootstrapped fossil *d* is 19.9 (s.d = 1.3).

species pair lake	mean *d*	*t* statistic	degrees of freedom	*p*-value	lake GPS coordinates
Little Quarry	26.9	−32.5	186	< 0.0001	49.663 N, 124.109W
Enos	18.6	5.7	177	< 0.0001	49.280 N, 124.156W
Paxton	16.1	23.4	176	< 0.0001	49.708 N, 124.525W
Priest	12.1	39.1	194	< 0.0001	49.745 N, 124.565W
Emily^a^	7.8	65.4	195	< 0.0001	49.746 N, 124.545W

^a^Emily Lake is several hundred metres downstream from Priest Lake, so gene flow from Priest to Emily is possible. Emily Lake has been treated as non-independent (e.g. [6]), and independent (e.g. [60]). Removing the Emily Lake mean *d* from the remaining four species pairs changes the grand mean *d* to 18.3.

### (d) Timing of morphological divergence

Multivariate *d* between the first lineage II fossil sample and each subsequent fossil sample exceeded *d* of the least divergent *G. aculeatus* species pair after approximately 3100 years (approx. 1550 generations, assuming 2 years per generation [41]*.* Fossil *d* consistently surpassed mean species-pair *d* after approximately 8500 years (approx. 4250 generations). It reached its maximum by approximately 15 700 years (approx. 7850 generations), near the end of the sequence (figure 3). This suggests that further divergence could have occurred had stickleback not gone locally extinct [23].

### (e) Correlated trait evolution

Most traits showed moderate to highly correlated reduction from the first to the last lineage II sample (electronic supplementary material, figure S1; table 1). The average pairwise correlation between trait-mean vectors was 0.59 (s.d. = 0.33; median = 0.72; electronic supplementary material, figure S5). We also note that the evolutionary lag in pelvic score reduction is evidence for *in situ* evolution in this population as it is consistent with a wait for a *de novo*, loss-of-function mutation in a pelvic-specific *Pitx1* promotor [26]. If evolution was being driven by hybridization via immigration of low-armoured forms from elsewhere, that lag should have been shorter.

## 4. Discussion

We propose that the lineage II fossil stickleback sequence represents the initial stages of speciation that Darwin had argued would be present in the history of life but absent from a fragmentary fossil record. Gradual evolution generated ancestor-descendant divergence in lineage II *G. doryssus* that was greater than divergence observed in four of the five *G. aculeatus* species pairs (figure 3). Given that species-pairs divergence was likely enhanced by ecological character displacement [6], such divergence in this single-population fossil lineage is especially striking. Indeed, observed fossil divergence was qualitatively as extensive as any observed in the threespine stickleback adaptive radiation [61]. We therefore infer that the last, low-armoured lineage II population had evolved at least some reproductive isolation from its high-armoured, parapatric source.

This conclusion assumes that multivariate phenotypic divergence during local adaptation is indicative of reproductive isolation. Ecological speciation theory suggests that multivariate trait divergence generates reproductive isolation because trait divergence reflects adaptation to different environments. Adaptation increases potential for mating barriers by making it more likely that hybrids would be unfit due to ecological inferiority to parental forms [62,63]. Empirically, stickleback species-pair ecotypes are adaptively divergent in size and shape for many traits used for feeding, swimming and defense [13,17]. Benthic-limnetic hybrids feed poorly because prey types and sizes suitable to intermediate hybrid trait values are rare; thus, hybrids grow more slowly and have low fitness [19–21], indicative of post-zygotic isolation [16]. These facts suggest a strong correlation between morphological divergence and reproductive isolation in stickleback.

In the fossils, the last sample of lineage II *G. doryssus* was significantly smaller in body size than the first sample (figure 2; electronic supplementary material, figure S1); body size is a known correlate of mate choice and assortative mating in the species pairs [7,47,48]. Moreover, the first and last samples of *G. doryssus* lineage II differed significantly for 11 other traits related to feeding, swimming and defense (figure 2 and table 1). This divergence likely reflects local adaptation to novel conditions that existed in the depositional environment, including shallower water [23,39,40], fewer piscivorous vertebrates [23] and different prey [38]. Diet and predator variation among habitats cause divergent natural selection in *G. aculeatus* [64,65]. Thus, we conclude that our morphological proxy is reliable evidence of early ecological speciation in *G. doryssus*.

This conclusion is supported by the observation that intermediate forms were rarely observed whenever high- and low-armour forms were found together in lineage I samples. That is, instances of co-occurrence between high- and low-armoured *G. doryssus* individuals were not accompanied by increased frequencies of putative hybrids, indicating that reproductive isolation from the parapatric source accompanied morphological divergence by late lineage II samples.

What does this system say about the mode, pace and permanence of speciation? First, evidence for reproductive isolation potentially changes the interpretation of evolution in this system from one of ‘anagenetic’ phyletic evolution (e.g. [66]) to one of ‘punctuated cladogenesis', wherein a peripheral population evolved reproductive isolation while its ancestral population persisted [42].

Second, these data reveal the order, timing and covariance of evolution by multiple traits during initial speciation. Visual comparison of trait evolution suggests that armour traits diverged first and fastest before levelling off (electronic supplementary material, figure S1). By contrast, reductions in non-armour traits and body size were slower and more linear. Reductions in means tended to be highly correlated through time for most traits (electronic supplementary material, figure S5), suggesting either selection acting on multiple parts of the phenotype independently, or selection on a few traits that caused evolution of genetically correlated phenotypes.

Third, the fossils show that reproductive isolation can evolve rapidly, fitting ecological speciation models for peripheral populations adapting to novel environments [67,68]. In *G. doryssus*, inferred reproductive isolation between high- and low-armoured forms accrued over several thousand generations (figure 3), consistent with time estimated available for the species pairs and other post-glacial ecotype pairs [7,13,69–71]. Palaeobiologically, such rapid divergence underscores how hard it is to capture speciation in the fossil record. Bell & Haglund [72], with only six samples from the same deposit, failed to detect the arrival of lineage II. Only sampling at approximately 5000-year intervals [24] (typically the lower limit of fossil temporal resolution [73]) captured the replacement event. Without this system's temporal scope and resolution, we would not have been able to infer the timing of reproductive isolation, since lineage I and the later, low-armoured samples from lineage II are phenotypically similar.

Notably, in the past few decades, phenotypic divergence, genomic divergence and size-based positive assortative mating in *G. aculeatus* has evolved between anadromous ancestors and newly founded freshwater descendants. This divergence is comparable to that between anadromous and old freshwater populations [55,74]. If species-level phenotypic divergence can evolve so quickly, why did it take thousands of generations for lineage II stickleback to diverge? The answer may be that freshwater *G. aculeatus* mostly use low-frequency standing genetic variation during freshwater adaptation [32,54,55,74–76], so divergence can proceed immediately [77]. By contrast, slower divergence by lineage II *G. doryssus* may have resulted in part from lack of adaptive genetic variation. For example, pelvic skeletal reduction occurred about 3000 years later than reduction of several other armour traits [26], indicating a lack of genetic variation for pelvic reduction.

Fourth, morphological divergence and early speciation by lineage II were impermanent. Lineage II was replaced by a killifish at the top of the sequence, only approximately 16 000 years after its appearance [23]. Such disappearance is a major feature of the stickleback radiation, as most freshwater lineages have gone extinct during glacial cycling without spawning new species [78]. For examples, similar time to extinction has been observed in the post-glacial Hadley Lake stickleback species-pair after introduction of an exotic catfish [14], whereas the Enos Lake species pair recently collapsed following arrival of an invasive crayfish that altered habitat use and increased hybridization between benthics and limnetics [14,16,43,44]. For stickleback, the environments that favour diversification and initial stages of speciation are ephemeral, leading to rapid evolution and disappearance of stickleback species [79]. This generates an evolutionary history resembling a ‘phylogenetic raceme’ [80–82].

The rapid disappearance of *G. doryssus* (and modern stickleback) novelty therefore supports one tenet of the ‘ephemeral speciation’ model proposed by Rosenblum *et al*. [83] in a synthesis of previous ideas. They noted that new species can accrue rapidly, on the order of hundreds of species per lineage per million years, evidenced by young adaptive radiations like Hawaiian *Drosophila* [84] and *Cyprinodon* pupfish [85]. However, estimates from phylogenies and the fossil record suggest typical rates closer to 1 to 10 species per lineage per million years [83]. The ephemeral speciation model reconciles this rate discrepancy by suggesting that young species often disappear without record because they tend to consist of small, geographically restricted, peripheral populations [67,86] that are prone to extinction or to reabsorption via hybridization with larger, geographically widespread parent populations.

Last, rapid divergence and disappearance of lineage II *G. doryssus* supports ‘ephemeral divergence’, Futuyma's mechanism for punctuated equilibrium [87,88]. Punctuated equilibrium is the observation that morphological evolution in the fossil record is concentrated at cladogenetic speciation events [4]. Following ideas of ‘genetic revolution’ [3,67], Gould advocated that punctuated equilibrium was the result of rare but major reorganizations of genetic architecture in small, peripheral populations (e.g. [89]). This mechanism, reminiscent of Goldschmidt's hopeful monsters ([90]; discussed in [42,89]), departed controversially from Neodarwinian gradualism (reviewed in [23,42] for *G. aculeatus*). Futuyma suggested instead that punctuational patterns could occur if peripheral populations evolved novelty (per [67]), but then regularly went extinct or were reabsorbed [87,88]. Only permanent speciation followed by range and demographic expansion could cement morphological novelty enough to be detected in the fossil record, linking morphological diversification to speciation without requiring non-Darwinian saltations ([42,87,88], pp. 79–82) where Gould confirms Futuyma's model). The rapid appearance, divergence and extinction of lineage II *G.doryssus* in Quarry D requires only standard Neodarwinian gradualism.

## 5. Conclusion

Darwin [2] attributed the absence of species transitions in the fossil record to the likelihood that speciation would proceed in small, isolated populations, making fossilization and discovery unlikely. Mayr's [67,91] peripatric model of speciation added that speciation in peripheral isolates could be fast, and the original punctuated equilibrium [4] was simply Mayr's model applied to the fossil record. Multivariate divergence in lineage II *G. doryssus* appears to follow these models, occurring after colonization of a peripheral habitat in an isolated lake basin [23] more quickly than can be resolved by most fossil sequences [5,73,92]. Comparison to replicated, extant, reproductively isolated pairs of benthic-limnetic ecotypes suggests that fossil divergence was sufficient to generate reproductive isolation. However, the impermanence of both lineage I and lineage II low-armoured *G. doryssus* underscores that young stickleback species often go extinct. These findings support models that explain morphological stasis in the fossil record [4,87], and that reconcile variable speciation rates estimated on different time scales [83]. And our findings help to solve the paradox that there are so few species within the Gasterosteidae despite the high evolvability and adaptability that make stickleback a model system in evolutionary ecology [61,81,93].

## Data Availability

Code and data to recreate analyses are at https://doi.org/10.5061/dryad.mpg4f4r53 [53]. Supplementary material is available online [94].
